# *Bojesodok-eum*, a Herbal Prescription, Ameliorates Acute Inflammation in Association with the Inhibition of NF-**κ**B-Mediated Nitric Oxide and ProInflammatory Cytokine Production

**DOI:** 10.1155/2012/457370

**Published:** 2012-10-08

**Authors:** Kook Ho Sohn, Mi Jeong Jo, Won Joon Cho, Jong Rok Lee, Il Je Cho, Sang Chan Kim, Young Woo Kim, Seon Young Jee

**Affiliations:** ^1^Department of Ophthalmology, Otolaryngology and Dermatology, College of Oriental Medicine, Daegu Haany University, Daegu 706-828, Republic of Korea; ^2^Medical Research Center for Globalization of Herbal Formulation and College of Oriental Medicine, Daegu Haany University, Daegu 706-828, Republic of Korea; ^3^Department of Herbal Pharmaceutical Engineering, Daegu Haany University, Kyung-San, Kyung-buk 712-715, Republic of Korea

## Abstract

*Bojesodok-eum* (BSE) is a herbal prescription consisting of *Coptidis Rhizoma* and *Scutellariae Radix* as main components. This paper investigated the effects of BSE on the induction of nitric oxide (NO), prostaglandin E_2_ (PGE_2_), and proinflammatory cytokines that are caused by lipopolysaccharide (LPS) in murine macrophage cell line and on the paw edema formation in animals. Administration of BSE (0.3 g/kg and 1 g/kg) in rats significantly inhibited carrageenan-induced paw edema formation, as did dexamethasone, an anti-inflammatory positive control drug. In cell model, treatment of BSE decreased the production of NO and PGE_2_ in RAW264.7 cells stimulated by LPS. BSE also inhibited the expression of iNOS and COX-2 protein as well as COX activity in a concentration-dependent manner. Consistently, BSE suppressed the ability of LPS to produce TNF-*α*, interleukin-1*β*, and interleukin-6. LPS treatment induced nuclear NF-*κ*B level and I-*κ*B*α* phosphorylation, which were inhibited subsequent treatment of BSE, suggesting its repression of LPS-inducible NF-*κ*B activation. BSE abrogated the induction of NO, PGE_2_, and proinflammatory cytokines, as well as iNOS and COX-2 protein expression in RAW264.7 cells stimulated by LPS as mediated with NF-*κ*B inhibition.

## 1. Introduction

Over the last few decades, medical herb has been prescribed with other herbs, which is called as herbal formula. Herbal prescription made by combination of medical herbs is essential for both potentiating efficacy and reducing toxicity in traditional oriental medicine. *Bojesodok-eum* (BSE) is one of the most frequently used prescriptions for the treatment of fever, jaundice, edema, and inflammatory disease and originated from *“Li Dongyuan's Effective Prescriptions” *(1180-1252, China), a famous classic in oriental medicine. It is composed of 14 medical herbs including *Coptidis Rhizoma *and *Scutellariae Radix *as main components. *C. Rhizoma *and *S. Radix *have been studied to treat inflammation and tissue injury and have various beneficial effects such as cancer chemoprevention. In recent studies, *C. Rhizoma *and *S. Radix*, the two main constituents of BSE, cooperatively had inhibitory effects on azoxymethane-induced aberrant crypt foci formation in rat colon and theophylline-induced increase in arterial blood pressure of rats [1, 2]. Nevertheless, the scientific proof and mechanistic basis for the anti-inflammatory effect of BSE have almost not been elucidated.

Inflammatory response is mainly regulated by nitric oxide (NO), eicosanoids, and cytokines, all of which are released by injured or infected cells and have important roles in progression of inflammatory states such as edema, intra/intercellular stress, and tissue necrosis [3]. Especially, proinflammatory cytokines such as tumor necrosis factor (TNF)-*α* and interleukin (IL) that are responsible for communication between immune cells have been widely accepted for involvement of promoting inflammatory processes [4, 5]. Lipopolysaccharide (LPS) is a gram-negative bacterial endotoxin and the first stimulus of sepsis [6]. LPS can directly activate inflammatory cells such as macrophages, which produce inflammatory mediators such as NO, eicosanoids, and cytokines [7, 8]. In particular, a large amount of NO and prostaglandin E_2_ (PGE_2_) was produced by inducible NO synthase (iNOS) and cyclooxygenase-2 (COX-2), respectively. They are the main factor that causes the harmful effects in the tissue [9]. 

Nuclear factor-kappa B (NF-*κ*B) is a key transcription factor to transactivate many genes related to the regulation of immune responses, cell adhesion and survival [10–12]. In the process of inflammation, NF-*κ*B transcriptionally activates iNOS and COX-2 and various inflammatory cytokines such as TNF-*α* and ILs [13, 14]. Reversely, TNF-*α* and IL-1*β* also activate NF-*κ*B to exaggerate the initial inflammatory responses [15]. It is well known that drug candidate involving anti-inflammatory effects suppresses cytokines production by inhibiting NF-*κ*B transactivation [16]. Nevertheless, present therapies to regulate inflammation remain to be further investigated.

In view of the importance of NO and the inflammatory cytokines in the process of inflammatory disorders and the need of a new drug for the treatment of inflammation, this study investigated the potential inhibitory effect of BSE on LPS-dependent iNOS and COX-2 expression as well as TNF-*α*, IL-1*β*, and IL-6 induction in macrophages. Furthermore, this study identifies BSE as a component with the inhibitory effects on paw edema formation in a rat model of acute inflammation. In terms of various applications of BSE in traditional medicine and its therapeutic potential effect, these findings certificate the crucial pharmacology of BSE and confirm the possibility of its therapeutic agent by offering scientific evidence.

## 2. Methods

### 2.1. Preparation of the Extract of BSE

 The herbal components of BSE were purchased from Daewon pharmacy (Daegu, Republic of Korea) (Table 1). BSE was prepared by boiling 226 g of BSE in 1 L of water for 3 h. The extract was filtered through a 0.2 *μ*m filter (Nalgene, New York, NY, USA), lyophilized, and stored at −20°C until use. The amount of BSE was estimated by the dried weight of lyophilized BSE. The yield of lyophilized BSE was 19.75%. 

### 2.2. Materials

Horseradish peroxidase-conjugated goat anti-rabbit, anti-mouse, and anti-goat IgGs were purchased from KPL (Gaithersburg, MD, USA). Anti-phospho-I-*κ*B*α* (p-I-*κ*B*α*), anti-NF-*κ*B p65, and Lamin A antibodies were supplied from Santa Cruz Biotechnology (Santa Cruz, CA, USA). Anti-COX-2 and COX-1 antibodies were from Cell Signaling (Beverley, MA, USA) and Cayman (MI, USA), respectively. Antimurine iNOS antiserum was purchased from Transduction Laboratories (Lexington, KY). Polyethylene glycol #400 (PEG) solution was obtained from Yakury Pure Chemical Co. (Kyoto, Japan). Carrageenan, dexamethasone, standard compounds in BSE, and other reagents were purchased from Sigma Chemical Co. (St. Louis, MO, USA).

### 2.3. Animal Experiment

Animal experiments were conducted under the guidelines of the Institutional Animal Care and Use Committee (IACUC) at Daegu Haany University [17]. Sprague-Dawley rats at 6 weeks of age (male, 140–160 g) were provided from Samtako Co. (Osan, Korea), acclimatized for 1 week, and maintained in a clean room at the Animal Center for Pharmaceutical Research, College of Oriental Medicine, Daegu Haany University. Animals were caged under the supply of filtered pathogen-free air, commercial rat chow (Purina, Korea), and water ad libitum at a temperature between 20 and 23°C with 12 h light and dark cycles and relative humidity of 50%. 

### 2.4. Carrageenan-Induced Paw Edema

Sprague-Dawley rats (*N* = 24) were randomly divided into four groups, and thus each group consisted of six animals. BSE, dissolved in 40% PEG, was orally administered to rats at the dose of 0.3 or 1 g/kg/day for 4 days. Dexamethasone (1 mg/kg/day), an anti-inflammatory drug, was used as a positive control [18]. To induce acute inflammation in paw, rats were injected into the hind paw with a 1% solution of carrageenan (s.c.) dissolved in saline after vehicle or BSE treatment. The paw volumes were measured up to 4 h after the injection at intervals of 1 h. The hind paw volume was determined volumetrically by measuring with a plethysmometer (Letica, Rochester, MI, USA). After euthanasia using ether, the hind paw samples were collected. 

### 2.5. Histological Process

The hind paw skins—dorsum and ventrum pedis skins—were separated and fixed in 10% neutral buffered formalin, then embedded in paraffin, sectioned (3~4 *μ*m), and stained with hematoxylin and eosin (H&E) [19]. The histopathological profiles of each sample were observed under light microscope (Nikon, Japan). 

### 2.6. Histomorphometry

 The thicknesses of dorsum pedis and ventrum pedis skins (from epidermis to dermis; keratin layers were excluded) were measured using automated image analyzer (DMI-300 Image Processing; DMI, Korea) under magnification 40 of microscopy (Nikon, Japan) at prepared skin histological samples as mm/paw.

### 2.7. RNA Preparation and Real-Time Polymerase Chain Reaction (PCR) Assays

Total RNA was extracted from the macrodissected formalin fixed paraffin embedded (FFPE) samples with the RNeasy FFPE kit (Qiagen, Tokyo, Japan) following the manufacturer's instructions [20]. Real-time PCR was carried out according to the manufacturer's instructions by using a Light CyclerDNA master SYBR green-I kit (Light-Cycler 2.0, Roche, Mannheim, Germany). The relative levels of iNOS and COX-2 were normalized based on the level of glyceraldehyde-3-phosphate dehydrogenase. After PCR amplification, a melting curve of each amplicon was determined to verify its accuracy. 

### 2.8. Cell Culture

 RAW264.7 cell, a murine macrophage cell line, was obtained from American Type Culture Collection (Rockville, MD, USA). The cells were maintained in Dulbecco's modified Eagle's medium (DMEM) containing 10% fetal bovine serum (FBS), 50 U/mL penicillin, and 50 mg/mL streptomycin at 37°C in a humidified atmosphere with 5% CO_2_. For all experiments, the cells were grown to 80–90% confluency and were subjected to no more than 20 cell passages. RAW264.7 cells were incubated with 1 *μ*g/mL LPS (Escherichia coli 026:B6; Sigma, St. Louis, MO, USA). The cells were incubated in the medium without 10% FBS for 12 h and then exposed to LPS or LPS + BSE for the indicated time periods. BSE as dissolved in dimethylsulfoxide was added to the incubation medium 1 h prior to the addition of LPS.

### 2.9. MTT Cell Viability Assay

The cells were plated at a density of 5 × 10^4^ cells per well in a 96-well plate to determine any potential cytotoxicity [21]. Cells were serum-starved for 12 h and then treated with BSE for the next 24 h. After incubation of the cells, viable cells were stained with MTT (0.5 *μ*g/mL, 4 h). The media were then removed, and produced formazan crystals in the wells were dissolved by addition of 200 mL dimethylsulfoxide. Absorbance was measured at 540 nm using a Titertek Multiskan Automatic ELISA microplate reader (Model MCC/340, Huntsville, AL, USA). Cell viability was defined relative to untreated control cells (i.e., viability (% control) = 100 × (absorbance of treated sample)/(absorbance of control)).

### 2.10. Assay of Nitrite Production

NO production was monitored by measuring the nitrite content in culture medium [17]. This was performed by mixing the samples with Griess reagent (1% sulfanilamide, 0.1% N-1-naphthylenediamine dihydrochloride, and 2.5% phosphoric acid). Absorbance was measured at 540 nm after incubation for 10 min.

### 2.11. Enzyme-Linked Immunosorbent Assay (ELISA)

RAW264.7 cells were preincubated with BSE for 1 h and continuously incubated with LPS for 24 h [17]. PGE_2_, TNF-*α*, IL-1*β*, and IL-6 contents in the culture medium were measured by ELISA using anti-mouse PGE_2_, TNF-*α*, IL-1*β*, or IL-6 antibodies and biotinylated secondary antibody according to the manufacturer's instruction (Endogen, Woburn, MA, USA).

### 2.12. Immunoblot Analysis

Cells were lysed in the buffer containing 20 mM Tris*·*HCl (pH 7.5), 1% Triton X-100, 137 mM sodium chloride, 10% glycerol, 2 mM EDTA, 1 mM sodium orthovanadate, 25 mM b-glycerophosphate, 2 mM sodium pyrophosphate, 1 mM phenyl methyl sulfonyl fluoride, and 1 mg mL-1 leupeptin [22]. Cell lysates were centrifuged at 10,000 g for 10 min to remove debris. Proteins of interest were visualized using 5-bromo-4-chloro-3-indolylphosphate and 4-nitroblue tetrazolium chloride or ECL chemiluminescence detection kit. Equal loading of proteins was verified by actin immunoblottings. Repeated experiments were separately performed to confirm changes.

### 2.13. Scanning Densitometry

Scanning Densitometry of the immunoblots was performed with an Image Scan & Analysis System (Alpha-Innotech, San Leandro, CA, USA). The area of each lane was integrated using the software Alpha EaseTM version 5.5 (Alpha-Innotech) followed by background subtraction.

### 2.14. COX Activity Analysis

COX enzyme activity was measured using ELISA kit. The treated RAW264.7 cells were collected by centrifugation at 1,500 ×g for 10 min at 4°C and homogenized in cold buffer (0.1 M Tris-HCl, pH 7.8, 1 mM EDTA, 250 mM mannitol, and 0.3 mM diethyldithiocarbamic acid). Homogenized cells were centrifuged at 10,000 ×g for 15 min at 4°C, and the supernatant was removed for COX activity assay. Protein content of supernatant was estimated according to Bicinchoninic acid (BCA), and the cell lysate was reacted with colorimetric substance, N-N-N′-N′-tetramethyl-p-phenylenediamine (TMPD), and arachidonic acid for 5 min at 25°C. This kit can measure the peroxidase activity of COX. The peroxidase activity was assayed colorimetrically at 590 nm. 

### 2.15. Profiling the Chemical Contents of BSE by UPLC

The UPLC (ultraperformance liquid chromatography) system (Waters, USA), which was equipped with a pump Waters ACQUITY ultraperformance LC system (USA) and a Waters ACQUITY photodiode array detector (PDA), was used for analysis. The empower data system was used for recording of the output signal of the detector. Separation was executed on a Waters ACQUITY BEH C18 column (1.7 **μ**m, 2.1 *× *100). The mobile phase was composed of water and acetonitrile at a flow rate of 0.4 mL/min. The injection volume was 2 **μ**L. The detection UV wavelength was set at 277 and 280 nm. Standard stock solutions of four marker components, baicalin, baicalein, berberine and wogonin, were prepared by dissolving at a concentration of 100 **μ**g/mL in 10ml of methanol and diluted with methanol for production of working standard solutions [23].

### 2.16. Statistical Analysis

One-way analysis of variance (ANOVA) was used to assess statistical significance of differences among treatment groups. For each statistically significant effect of treatment, the Newman-Keuls test was used for comparisons between multiple group means. The data were expressed as means ± 95% confidence intervals (CI). All statistical tests were two-sided.

## 3. Results

### 3.1. Inhibition of Carrageenan-Induced Acute Inflammation by BSE

**Figure 1:**
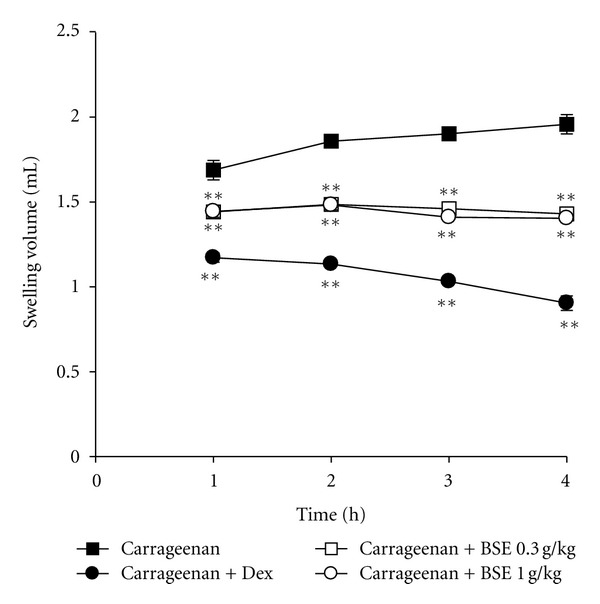
Inhibition of carrageenan-induced paw edema formation by BSE. BSE was administered to rats at the oral dose of 0.3 or 1 g/kg/day. Then, paw edema was induced by subcutaneously injecting 1% solution of carrageenan dissolved in saline (0.1 mL per animal) into the hind paw. The thickness of the paw was measured before and 1–4 h after carrageenan injection. Dexamethasone (Dex, 1 mg/kg, p.o.) was used as a positive control. Data represents the mean ± S.E.M. of six animals (significant as compared with carrageenan alone, **P < 0.01). For data points where error bars could not be seen, the standard error was subtended by the data point. BSE: *Bojesodok-eum. *

**Figure 2:**
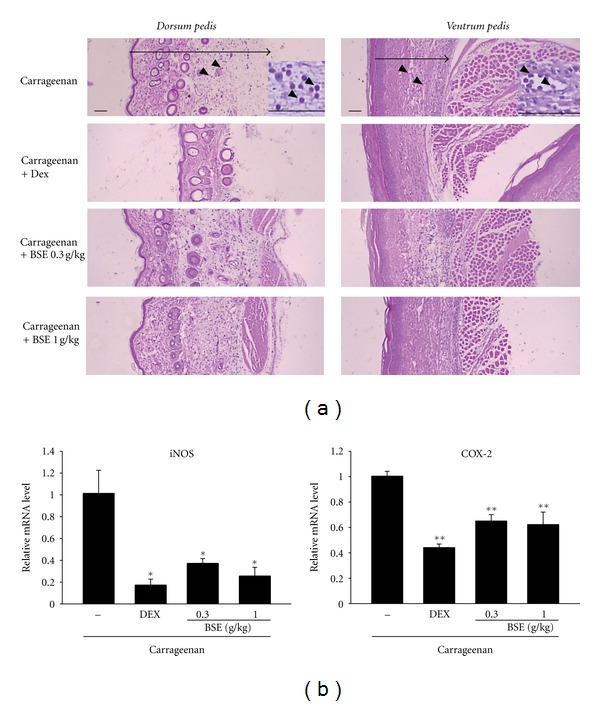
Changes on histological profiles and proinflammatory genes in the paw. (a) In each rat, cutaneous regions of *dorsum* and *ventrum pedis* were stained with H&E and used for histological sample preparation in this study. Arrow indicated total thicknesses measured, and arrow heads were infiltrated inflammatory cells. Scale bars = 160 *μ*m. Inset of upper picture is the macrophotography to show more precisely the infiltration of inflammatory cells. (b) The levels of mRNAs in the paw of rats analyzed by real-time PCR. Values represent the mean ± S.E.M. (significantly different as compared to carrageenan-treated group, *P < 0.05, *P < 0.01). BSE: *Bojesodok-eum. *

 First, we determined the inhibitory effects of BSE on acute inflammation *in vivo*. We used the carrageenan-induced paw edema model, which is one of the widely used models for screening the efficacy of anti-inflammatory drugs [24]. Paw edema formation by carrageenan was observed from 1 h and persisted up to 4 h after injection (Figure 1). Administrations of BSE (0.3 and 1 g/kg) significantly inhibited the ability of carrageenan to induce paw swelling. We also showed the inhibitory effect of dexamethasone, a positive control, on edema formation. In addition, we confirmed the effects of BSE on histological profiles of *dorsum *and *ventrum pedis* skin. The changes of histomorphometrical analysis of hind paw skins were listed in Table 2. Marked increases of skin thicknesses on both *dorsum* and *ventrum pedis* were detected by treatment of carrageenan, which was blocked by BSE treatments (Figure 2(a)). Moreover, iNOS and COX-2 are crucial enzymes in the pathological process of acute inflammation. We assessed the expression of iNOS and COX-2 in the paw by real-time PCR. BSE treatment significantly prevented the iNOS and COX-2 induction by carrageenan injection in rats (Figure 2(b)). These results suggest that BSE suppresses the acute phase of paw swelling in association with inflammation *in vivo*. 

### 3.2. Inhibition of LPS-Inducible NO and PGE_2_ by BSE

**Figure 3:**
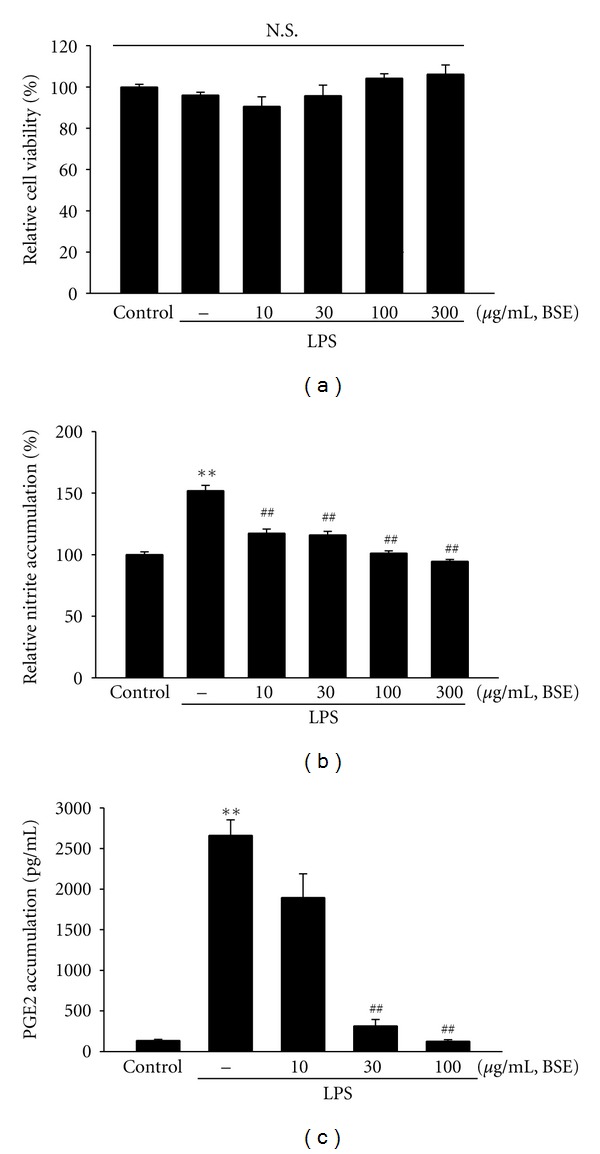
Inhibition of LPS-inducible NO and PGE_2_ by BSE. (a) MTT assay. RAW264.7 cells were treated with 10, 30, 100, and 300 *μ*g/mL BSE for 1 h and continuously incubated with LPS (1 *μ*g/mL). (b) NO and (c) PGE_2_ productions. RAW264.7 cells were treated with BSE at the indicated concentration for 1 h and continuously incubated with LPS (1 *μ*g/mL) for the next 24 h. NO and PGE_2_ concentration in culture media was monitored, as described in the Methods section. Data represents the mean ±S.E.M. from three separate experiments (significant as compared with vehicle-treated control, **P < 0.01; significant as compared with LPS alone, ^##^ *P* < 0.01). N.S. not significant; BSE: *Bojesodok-eum. *

 This study was extended to verify anti-inflammatory effect of BSE in a cell model. To test cellular toxicity of BSE, MTT assay was assessed in RAW 264.7 cells.  Figure 3(a) showed that cell survival was not affected by BSE treatment up to 300 *μ*g/mL. Next, we assessed the effect of BSE (10–300 *μ*L/mL) on NO and PGE_2_ production in RAW264.7 cells. NO and PGE_2_ productions were measured in the media of RAW264.7 cells treated with LPS and/or BSE as described in Method section. LPS treatment for 24 h increased NO production by 160% compared to control, which was inhibited by treatment of BSE (Figure 3(b)). In the subsequent experiments, we chose 10–100 *μ*g/mL concentrations of BSE to verify its effect on PGE_2_ production. BSE treatment markedly blocked PGE_2_ production in RAW 264.7 cell stimulated with LPS (Figure 3(c)).

### 3.3. Inhibition of LPS-Inducible TNF-*α*, IL-1*β*, and IL-6 by BSE

**Figure 4:**
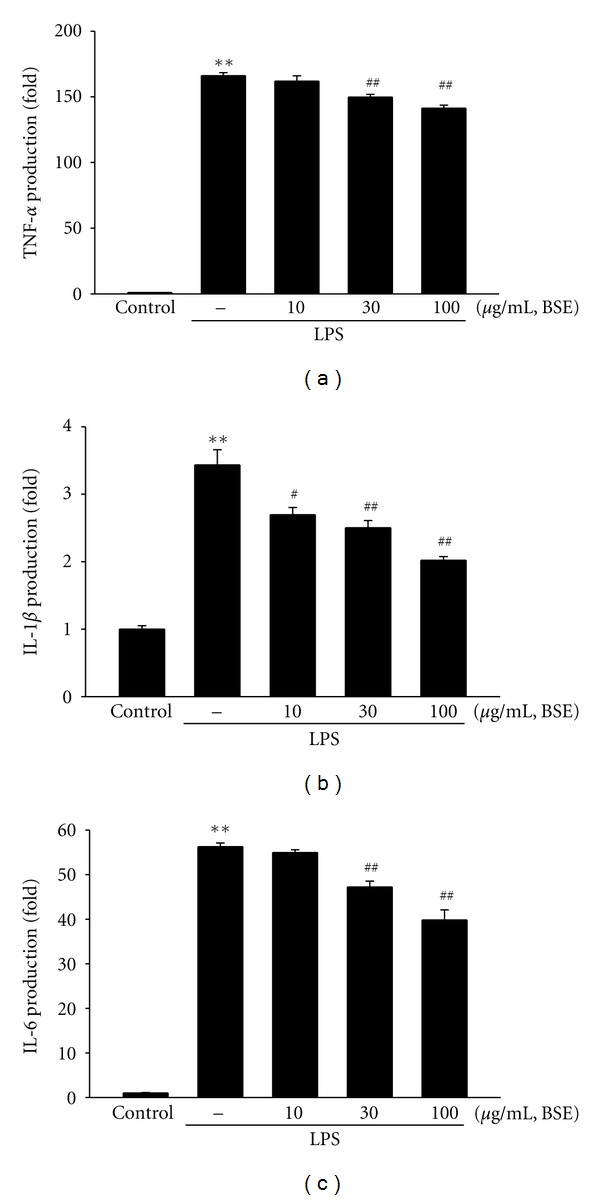
Inhibition of LPS-inducible TNF-*α*, IL-1*β*, and IL-6 by BSE. (a) TNF-*α*, (b) IL-1*β*, and (c) IL-6 contents in culture medium. RAW264.7 cells were treated with 10, 30, and 100 *μ*g/mL BSE for the 1 h and continuously incubated with LPS (1 *μ*g/mL) for 24 h. Data represents the mean ± S.E.M. from three separate experiments (significant as compared with vehicle-treated control, **P < 0.01; significant as compared with LPS alone, ^##^ *P* < 0.05, ^##^ *P* < 0.01). BSE: *Bojesodok-eum. *

 Cytokines are the major regulator in inflammatory responses and released by injured or infected cells. Next, we verified the effects of BSE on proinflammatory cytokines including TNF-*α*, IL-1*β*, and IL-6. We measured production of the cytokines in the media of RAW264.7 cells by using ELISA assays. LPS stimulation significantly increased the production of the cytokines (Figures 4(a)–4(c)). Concomitant treatment of BSE blocked the ability of LPS to increase the proinflammatory cytokine productions. 

### 3.4. Inhibition of LPS Induction on iNOS and COX-2 by BSE

**Figure 5:**
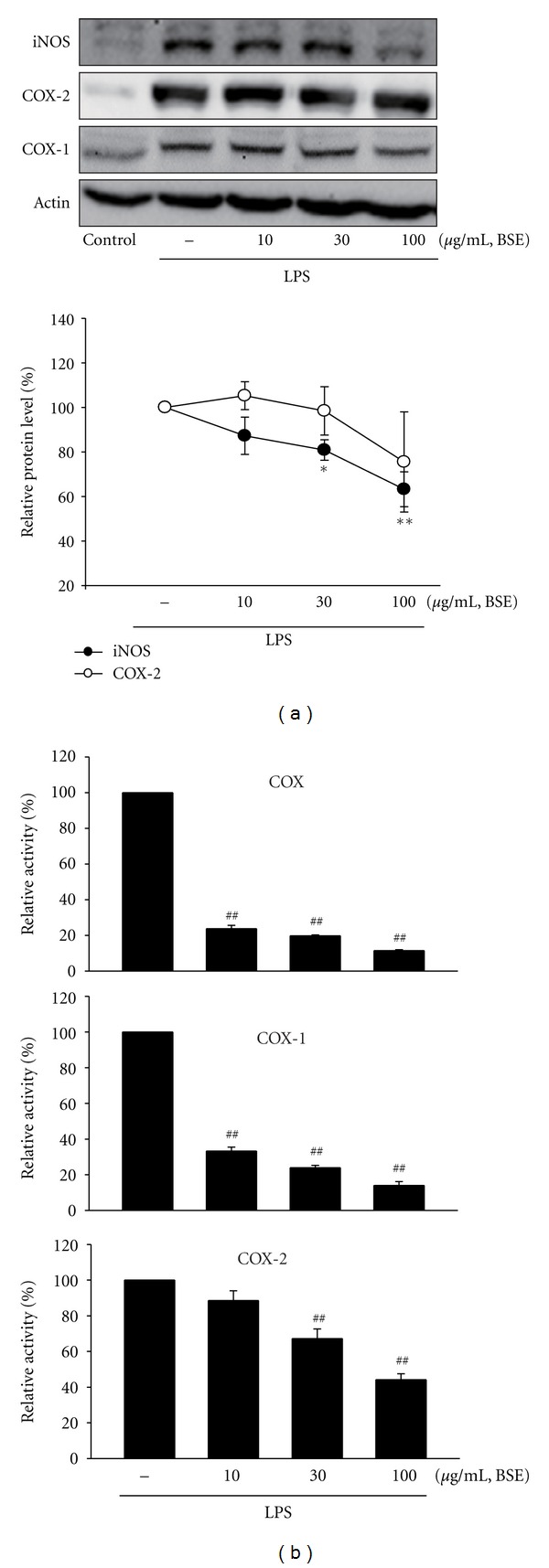
Inhibition of LPS-inducible iNOS and COX-2 by BSE. (a) iNOS and COX-2, immunoblottings (upper). iNOS, COX-2, or COX-1 protein levels were monitored 12 h after treatment with LPS (1 *μ*g/mL). Relative iNOS and COX-2 protein levels of the upper immunoblottings (lower). Values represent the mean ± S.E.M. (significantly different as compared to LPS-treated group, *P < 0.05, *P < 0.01). (b) Inhibition of LPS-inducible COX activity by BSE. The COX, COX-1, or COX-2 enzyme activity was measured as described in the Methods section. Data represents the mean ± S.E.M. from three separate experiments (significant as compared with LPS alone, ^##^ *P* < 0.01). BSE: *Bojesodok-eum. *

Next, we assessed the protein expression of iNOS and COX-2 by western blotting. LPS treatment markedly induced iNOS and COX-2, whereas BSE treatment (10–100 *μ*L/mL) prevented the iNOS and COX-2 induction (Figure 5(a), upper). BSE also inhibited COX-1 induction by LPS. Although BSE treatment almost completely abrogated LPS-induced iNOS, analysis using densitometer revealed that ability of BSE to inhibit COX-2 and COX-1 expressions was less potent than its effect on iNOS (Figure 5(a), lower). Therefore, we examined the effect of BSE on COX activity. Interestingly, treatment of BSE (10–100 *μ*L/mL) significantly inhibited LPS-inducible COX activity (Figure 5(b)). Moreover, COX-1 and COX-2 activity were also prevented by BSE treatment (Figure 5(b)). This data confirmed that BSE treatment inhibits iNOS and COX-2 expression as well as COX-2 enzyme activity. 

### 3.5. Inhibition of LPS-Inducible NF-*κ*B Activation by BSE

**Figure 6:**
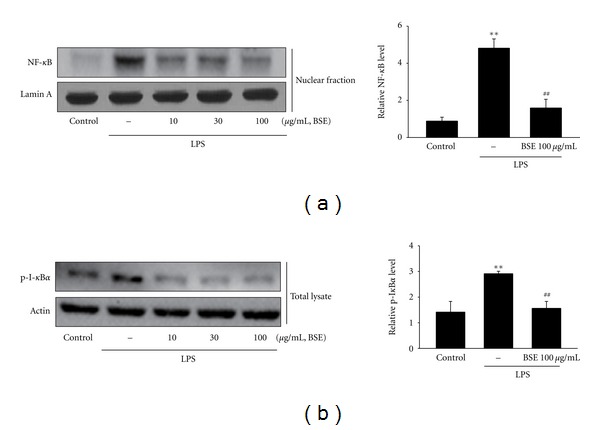
Inhibition of LPS-induced NF-*κ*B activation by BSE. (a) Nuclear NF-*κ*B protein level. Immunoblottings for lamin A verified equal loading and purity of the nuclear proteins. (b) Immunoblottings for phosphorylated I-*κ*B*α* (p-I-*κ*B*α*). The cells were treated with LPS or LPS + BSE for 1 h. Immunoblots are representative results from repeated experiments. For (a) and (b), values represent the mean ± S.E.M. (significant as compared with vehicle-treated control, **P < 0.01; significant as compared with LPS alone, ^##^ *P* < 0.01). BSE: *Bojesodok-eum. *

 NF-*κ*B is the key transcription factor for the inflammatory genes such as iNOS and COX-2 and activated in immune cells stimulated by LPS or other inflammatory challenges [10–12]. Translocation of NF-*κ*B to the nucleus is permitted by phosphorylation of I-*κ*B*α* and degradation of I-*κ*B*α* subunit. We then assessed the nuclear level of NF-*κ*B in the cells treated with LPS with or without BSE. The treatment of BSE in RAW 264.7 cells significantly inhibited LPS-inducible increase in nuclear level of NF-*κ*B (Figure 6(a)). Furthermore, exposure of LPS increased phosphorylation of I-*κ*B*α*, which was also blocked subsequent treatment of BSE (Figure 6(b)). Thus, BSE might prevent iNOS and COX-2 gene induction by inhibiting NF-*κ*B activation.

### 3.6. Effect of Baicalin and Berberine on Production of NO and PGE_2_

**Figure 7:**
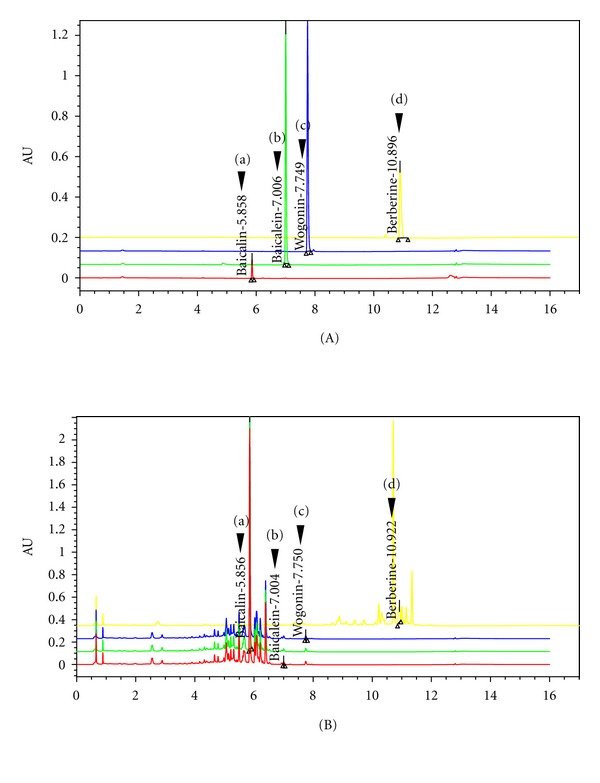
UPLC chromatogram of four marker compounds in BSE. (A) UPLC chromatogram of commercial standard compounds. (B) UPLC chromatogram of four marker compounds in BSE. The chromatograms were obtained at 277 nm (a, b, and c) and 280 nm (d). Baicalin (a), baicalein (b), wogonin (c), and berberine (d). BSE: *Bojesodok-eum. *

**Figure 8:**
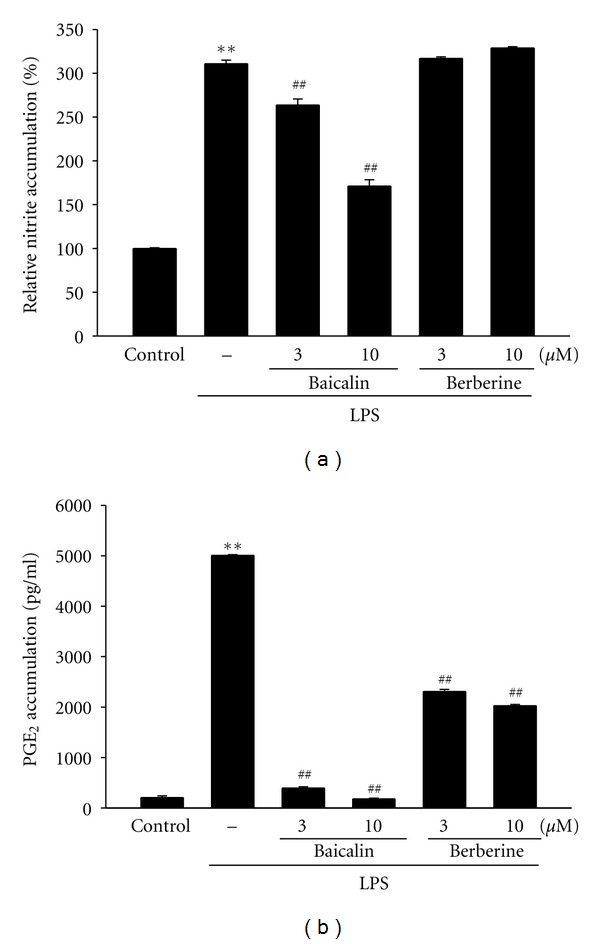
Inhibition of LPS-inducible NO and PGE_2_ by baicalin. (A) NO and (B) PGE_2_ productions. RAW264.7 cells were treated with baicalin or berberine at the indicated concentration for 1 h and LPS (1 *μ*g/mL) for the next 24 h. Data represents the mean ± S.E.M. from three separate experiments (significant as compared with vehicle-treated control, **P < 0.01; significant as compared with LPS alone, ^##^ *P* < 0.01).

*Coptidis Rhizoma *and *Scutellariae Radix *are two main herbs in the BSE. Therefore, we used the UPLC system in determination of four markers, baicalin, baicalein, berberine, and wogonin, which are the major components in *Coptidis Rhizoma *(i.e., berberine) and *Scutellariae Radix *(i.e., baicalin, baicalein and wogonin) [25, 26]. Contents of the four marker components were calculated from the calibration curve of the standards using UPLC (Table 3 and Figures 7(A) and 7(B)), indicating baicalin and berberine are most enriched compounds in BSE. Therefore, we determined the effects of baicalin, and berberine on the LPS-inducible NO and PGE_2_ accumulation in RAW264.7 cells. Baicalin treatment (3 and 10 *μ*M) markedly inhibited NO and PGE_2_ productions induced by LPS (Figures 8(a) and 8(b)). On the other hand, 3 and 10 *μ*M treatment of berberine failed to decrease in the NO induction, and, significantly, but less potently than baicalin, blocked PGE_2_ accumulation (Figures 8(a) and 8(b)). 

## 4. Discussion

Recently, it has been studied that carrageenan injection causes the release of NO as well as PGE_2_ in the peripheral tissue [27]. In edema formation, the NO and PGE_2_ might have a major function in development of hyperalgesia in response to inflammation and tissue injury. Furthermore, carrageenan is also known to induce the release of TNF-*α* and ILs in the tissue [28]. Here, we assessed that BSE administrations to rats markedly inhibited the induction of paw swelling by carrageenan. In H&E stating, BSE significantly decreased skin thicknesses on both *dorsum* and *ventrum pedis* induced by carrageenan. These results in this study demonstrate that BSE could inhibit the acute inflammation in rats. Next, we confirmed the effect of BSE on the production of NO and PGE_2_ in media of RAW264.7 cell stimulated by LPS. Pretreatment of BSE in the cells was significantly inhibited NO and PGE_2_ production. Also, we verified the effects of BSE on production of the cytokines, showing that BSE treatment markedly inhibited TNF-*α*, IL-1*β*, and IL-6 secretions induced by LPS. Furthermore, in view of BSE inhibition of paw edema formation and inflammatory cytokine production induced by LPS, the effects of BSE against edema formation might result from its inhibition of NO and TNF-*α* synthesis in the peripheral tissues. 

It has been shown that various signaling molecules and enzymatic pathways are involved in the process of production of inflammatory mediators. iNOS and COX-2 are key enzymes producing NO and PGs during inflammation, respectively [3, 29, 30]. Here, we investigated the level of iNOS and COX-2 protein levels. As a result of western blot analyses, BSE effectively blocked the inductions of iNOS and COX-2 proteins by LPS. Although BSE has marked efficacy on iNOS inhibition, its effect on COX-2 protein level was slight, but significant. Thus, we next examined COX enzyme activity. Interestingly, BSE treatment almost blocked the ability of LPS to induce COX, COX-1, and COX-2 enzyme activity in RAW 264.7 cells as well as iNOS and COX-2 expression induced by carrageenan in rats, indicating that the effects of BSE on the PGE_2_ production might result from its inhibition of COX enzyme activity. 

NF-*κ*B is a key transcription factor in the aspect of genes regulation related to inflammatory and immune responses, cell adhesion, and survival [10–12]. It has been shown that NF-*κ*B is interacted with its inhibitor protein, I-*κ*B*α*. Degradation of I-*κ*B*α* by its phosphorylation causes activation of NF-*κ*B and is subsequently translocated into the nucleus to transactivate target genes. We assessed immunoblot analysis to determine the effects of BSE on NF-*κ*B activation. Although LPS stimulated macrophages to induce I-*κ*B*α* phosphorylation and resultant increase in nuclear level of NF-*κ*B, pretreatment of BSE inhibited this process, showing that BSE might inhibit NF-*κ*B activation in association with BSE inhibition of I-*κ*B*α* phosphorylation. Moreover, it has been shown that I-*κ*B*α* is phosphorylated by I-*κ*B*α* kinase, which is activated by other kinases (e.g., protein kinase C and tyrosine kinase family members) [31, 32]. It is worthy of demonstrating the effects of BSE on these upstream kinases, and what a target protein of BSE that brings the anti-inflammatory effects is.

The BSE prescription is composed of 14 medical herbs including *Coptidis Rhizoma *and *Scutellariae Radix *as main constituents. The extract of *Coptidis Rhizoma *has been shown to inhibit activation of endotoxin-stimulated macrophage-like cells [33]. The *Scutellariae Radix* was also effective on PGE_2_ production and COX-2 expression [34]. Baicalin and berberine are the major component in *Scutellariae Radix *and* Coptidis Rhizoma, *respectively [25, 26]. This and previous studies found that baicalin, one of the most enriched compounds in BSE, was effective in the inhibition of NO and PGE_2_ productions induced by LPS [35]. On the other hand, although berberine (10 *μ*M) inhibited PGE_2_ accumulation, it failed to block in the NO induction, which was matched with previous findings (IC_50_ > 30 *μ*M) [36]. Therefore, baicalin might, at least in part, be one of the active compounds in BSE in the aspects of anti-inflammation in LPS-stimulated macrophages. 

## 5. Conclusions

In this study, we confirmed anti-inflammatory effects of BSE and used two different models: (1) an animal approach involving paw edema model in rats injected with carrageenan and (2) a cellular approach using RAW264.7 murine macrophage model treated with LPS. Based on these two models, we found that BSE had anti-inflammatory effects, which is related with its inhibition of NF-*κ*B activation in macrophages, thereby inhibiting the production of NO and proinflammatory cytokines. These findings showing the inhibition of paw swelling as well as inflammatory gene induction by BSE might help to understand the pharmacology and action mechanism of BSE. More importantly, we confirmed the existence of a novel candidate of anti-inflammatory traditional herbal prescription. This study also offers the possibility of treatment for inflammatory disease by traditional oriental medicine.

## Figures and Tables

**Table 1:** Herbal components in *Bojesodok-eum. *

Name of medical herbs	Percentage of contents in the prescription (%)
*Scutellariae Radix*	17.7
*Coptidis Rhizoma*	17.7
*Ginseng Radix*	10.7
*Aurantii Nobilis Pericarpium*	7.1
*Scrophulariae Radix*	7.1
*Glycyrrhizae Radix*	7.1
*Forsythiae Fructus*	3.5
*Arctii Fructus*	3.5
*Isatidis Radix*	3.5
*Lasiosphaeria*	3.5
*Bombycis*	2.2
*Cimicifugae Rhizoma*	2.2
*Bupleuri Radix*	7.1
*Platycodi Radix*	7.1

**Table 2:** Changes on the histomorphometrical analysis of hind paw skins.

Groups	*Dorsum pedis* skin (mm)	*Ventrum pedis* skin (mm)
Carrageenan	2.278 ± 0.155	1.285 ± 0.093
Carrageenan + Dexamethasone	0.828 ± 0.065**	0.844 ± 0.073**
Carrageenan + BSE 0.3 g/kg	2.050 ± 0.123*	1.144 ± 0.040*
Carrageenan + BSE 1 g/kg	1.928 ± 0.111**	1.096 ± 0.073*

Values are expressed as mean ± SD of 5 rat hind paws (significant as compared with carrageenan alone, *P < 0.05, **P < 0.01). BSE: *Bojesodok-eum.*

**Table 3:** Content of four marker components of BSE by UPLC.

Compound	Content (*μ*g/mL)
Baicalin	2194
Berberine	120
Baicalein	5.1
Wogonin	1.4

## Abbreviations

BSE: *Bojesodok-eum*
I-*κ*B: Inhibitor of *κ*B
IL: Interleukin
iNOS: inducible nitric oxide synthase
LPS: Lipopolysaccharide
NF-*κ*B: Nuclear factor-*κ*B
NO: Nitric oxide
PGE_2_: ProstaglandinE_2_
TNF-*α*: Tumor necrosis Factor-*α*.
